# An Investigation on the Electrochemical Behavior and Antibacterial and Cytotoxic Activity of Nickel Trithiocyanurate Complexes

**DOI:** 10.3390/ma13071782

**Published:** 2020-04-10

**Authors:** Amir M. Ashrafi, Pavel Kopel, Lukas Richtera

**Affiliations:** Faculty of Electrical Engineering and Communication, Department of Microelectronics, Brno University of Technology, Technicka 3058/10, CZ-616 00 Brno, Czech Republic; amirmansoor.ashrafi@mendelu.cz (A.M.A.); paulko@centrum.cz (P.K.)

**Keywords:** nickel complexes, trithiocyanuric acid, trimercaptotriazine, cyclic voltammetry

## Abstract

The electrochemical redox behavior of three trinuclear Ni(II) complexes [Ni_3_(abb)_3_(H_2_O)_3_(µ-ttc)](ClO_4_)_3_ (**1**), [Ni_3_(tebb)_3_(H_2_O)_3_(µ-ttc)](ClO_4_)_3_·H_2_O (**2**), and [Ni_3_(pmdien)_3_(µ-ttc)](ClO_4_)_3_ (**3**), where abb = 1-(1H-benzimidazol-2-yl)-N-(1H-benzimidazol-2-ylmethyl)methan-amine, ttcH_3_ = trithiocyanuric acid, tebb = 2-[2-[2-(1H-benzimidazol-2-yl)ethylsulfanyl]ethyl]-1H-benzimidazole, and pmdien = N,N,N′,N″,N″-pentamethyldiethylenetriamine is reported. Cyclic voltammetry (CV) was applied for the study of the electrochemical behavior of these compounds. The results confirmed the presence of ttc and nickel in oxidation state +2 in the synthesized complexes. Moreover, the antibacterial properties and cytotoxic activity of complex **3** was investigated. All the complexes show antibacterial activity against *Staphylococcus aureus* and *Escherichia coli* to different extents. The cytotoxic activity of complex **3** and ttcNa_3_ were studied on G-361, HOS, K-562, and MCF7 cancer cell lines. It was found out that complex **3** possesses the cytotoxic activity against the tested cell lines, whereas ttcNa_3_ did not show any cytotoxic activity.

## 1. Introduction

Trithiocyanuric acid (trimercaptotriazine acid, ttc) is an organic heterocyclic compound that consists of triazine ring and three sulfurs on carbon atoms. It can be either in thiol or thione form according to the conditions. As a trisodium salt, it is used for precipitation of heavy metals from wastewaters [1,2,3,4,5]. In the last three years, many papers on the utility of the acid appeared but the compound was mainly utilized for the preparation of nanostructures or nanocomposites with carbon nanomaterials [6,7,8]. Six viable donor atoms of ttc can be involved in the coordination to metal ions. Besides the precipitate formation, many compounds were prepared in crystalline form [9,10]. Metal ions can be coordinated by sulfur or nitrogen atom of ttc only to form mononuclear to trinuclear complexes; however, even more complicated compounds were prepared, such as hexanuclear gold(I) cluster [11] and sandwich-like structure of hexanuclear copper(I) complex [12]. Many of the important properties of the transition metals' complexes including their shape, color, magnetism, and reactivity depend on the electron occupancy of the metals'd-orbitals [13,14]. There are several parameters that can affect the geometrical structure of a transition metal's complex, particularly the number of d electrons of the metal. Some structures of mononuclear nickel(II) complexes have already been solved. The coordination sphere of nickel central atoms was completed by tridentate [15,16] or tetradentate nitrogen ligands [17,18,19]. Considering the properties of amine ligands, the coordination number of nickel can be either five or six with nitrogen only or N, S chelating mode of ttc. A later study on coordination properties of nickel ttc shows the possibility of preparing even heptanuclear cluster [Ni_7_(pmdien)_6_(H_2_O)_2_(µ-ttc)_3_](ClO_4_)_5_·3H_2_O [20].

Known platinum containing drugs including cisplatin [21] and oxaliplatin [22] show anti-cancer properties; however, platinum-based therapy lacks the selectivity for cancer cells and cause undesirable side effects such as nerve damage, hair loss, and nausea. Furthermore, platinum-based therapy is not effective against many common types of cancer. In addition, chemotherapy failure might be attributed to tumor resistance to oxaliplatin [23,24]. Therefore, it has been highly important to find potential substitutes for the platin drugs. In our previous work, we reported synthesis and study on mixed ligand complexes with Zn^2+^, Fe^2+^, and Mn^2+^ involving ttc ions [25]. It was found that iron and manganese ions show high cytotoxicity, whereas mononuclear zinc and nickel ones were inactive.

**Scheme 1:**
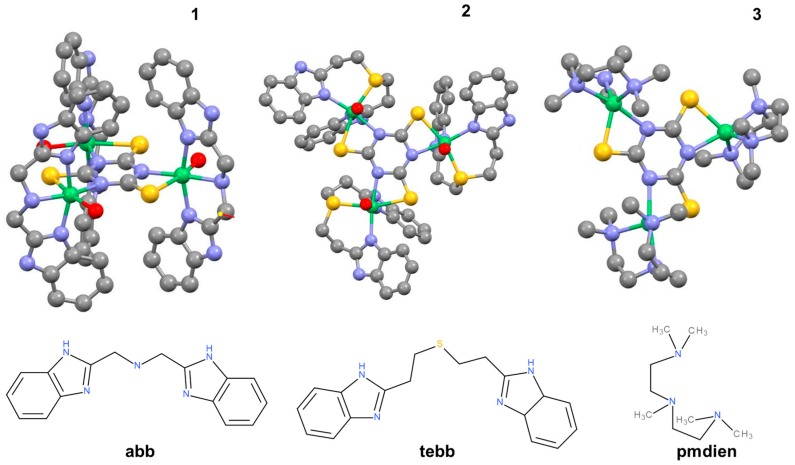
Trinuclear nickel(II) cations depicted with Mercury software [29]. Green stands for nickel, violet for nitrogen, yellow for sulfur, and red for oxygen atoms. Perchlorate anions and hydrogen atoms were omitted for clarity. The structural data of the complexes can be found in [26,27,28]. The ligands abb = 1-(1H-benzimidazol-2-yl)-N-(1H-benzimidazol-2-ylmethyl)methan-amine, tebb = 2-[2-[2-(1H-benzimidazol-2-yl)ethylsulfanyl]ethyl]-1H-benzimidazole, and pmdien = N,N,N′,N″,N″-pentamethyldiethylenetriamine are depicted under corresponding complexes.

The synthesis and structural characterization of three trinuclear Ni(II) complexes namely [Ni_3_(abb)_3_(H_2_O)_3_(µ-ttc)](ClO_4_)_3_ (**1**), [Ni_3_(tebb)_3_(H_2_O)_3_(µ-ttc)](ClO_4_)_3_·H_2_O (**2**), and [Ni_3_(pmdien)_3_(µ-ttc)](ClO_4_)_3_ (**3**), were previously reported [26,27,28] (Scheme 1). Here, we report on the electrochemical behavior of complexes that have not been studied yet. In our previous papers, we studied antibacterial and cytotoxic activities of complexes **1** and **2**, but such data were missing for complex **3**. It was our aim to complete the study on complex **3,** which contains methyl groups on pmdien ligand, and thus, hydrogen bonds are present in its structure [26]. 

## 2. Results and Discussion

### 2.1. Electrochemical Study

**Figure 1:**
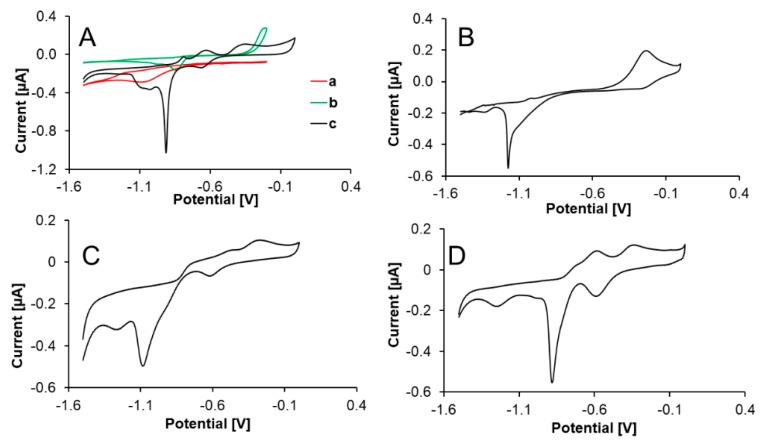
(**A**) Cyclic voltammogram of **a**: Blank (buffer composed of 1.750 mL H_2_O + 100 μL of the mixture (1 M HCl + 3 M NH_3_)), **b**: Ni^2+^ 250 ppb, **c**: ttc 5 × 10^−4^ M, (**B**) CV of (**1**), 5 × 10^−4^ M, (**C**) CV of (**2**), (**D**) CV of (**3**), 5 × 10^−4^ M. CV parameters: start potential −1.5 V, final potential 0.0 V, scan rate 50 mV·s^−1^.

**Scheme 2:**
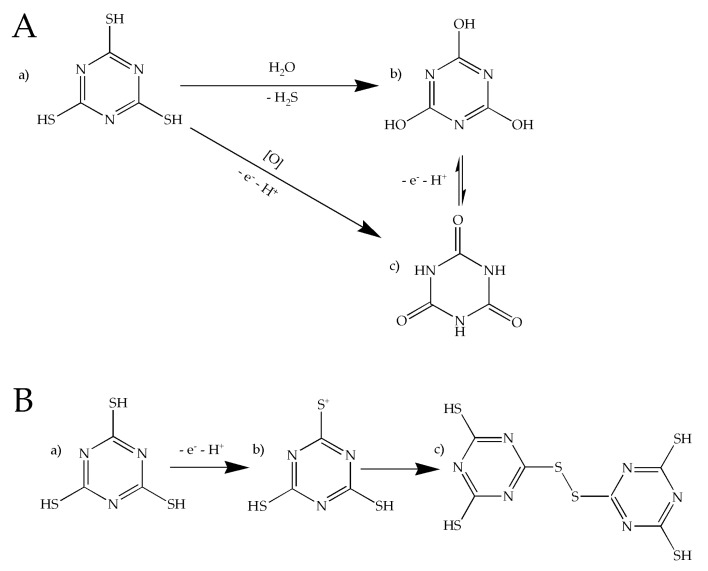
The proposed electrochemical redox mechanism of ttc. (**A**): two pathways of oxidation of ttc, (**B**): oxidation of ttc followed by the polymerization.

As shown in Figure 1A, there is a cathodic peak around −1.0 to −1.1 V, which appeared in all the recorded CVs. It is because of the reduction of the solvent component. In Figure 1A c, the CV of ttc shows three oxidation peaks at E_pa1_; −0.81 V, E_pa2_; −0.65 V, and E_pa3_; −0.40 V, respectively. There is also a reduction peak in cathodic scan at E_pc2_; −0.68 V. The electrochemical behavior of ttc is a complex process because of its structure. Its redox reaction might follow two main paths. First, the second oxidation and its corresponding reduction at E_pa2_; −0.65 V and E_pc2_; −0.68 V could be due to the reversible oxidation of tricyanic acid (Scheme 2A b), which can be formed by hydrolysis of ttc (Scheme 2A a). However, ttc itself can be irreversibly oxidized to s-triazine-2,4,6-trion (Scheme 2A c). This irreversible oxidation takes place at more positive potential (oxidation at E_pa3_; −0.40 V). In addition, the second mechanism corresponds to the oxidation peak at −0.81 V that may be due to the oxidation of ttc followed by the polymerization of the product (Scheme 2B). A similar mechanism has been previously reported for the melamine, which has structural similarities with ttc [30].

Furthermore, in Figure 1A b, an oxidation peak is observed at −0.22 V, which can be attributed to the oxidation of $Ni^{+2}\to Ni^{+3}$. The CVs of the studied complexes are shown in Figure 1B–D. In the case of complex **3,** the oxidation peaks can be observed at the same potentials as ttc. Thus, the presence of ttc in complex **3** is confirmed. The ligand pmdien is an aliphatic one and no other redox exchanges can be expected. Similarly, the oxidation peaks at the same potentials as ttc can be observed in the CV of complex **2,** indicating the existence of ttc in the complex. It is very probable that benzimidazole rings do not influence the electrochemical behavior of the complex due to the conformation of tebb ligands showing facial-like coordination with two nitrogen atoms of benzimidazoles in plane of octahedron and sulfur atom of benzimidazole in apical position causing twist of benzimidazole rings as reported before [28]. It is also possible that aliphatic chains in ligands do not allow for a simple exchange of electrons. However, the CV of complex **1** differs to the other complexes as only one reversible couple can be observed about −0.2 V, which might be due to the redox of the nickel. This Ni(II)–Ni(III) exchange might be promoted by planarity of ligand abb as well as by a smaller distance of benzimidazole moieties. Even though the oxidation potential of Ni^2+^/Ni^3+^ couple is highly affected by the composition of the complex, it was reported to vary from −0.24 to −0.42 V in complexes with thiolato groups [31,32], which is in accordance with the obtained results.

### 2.2. Antibacterial Activity 

**Figure 2:**
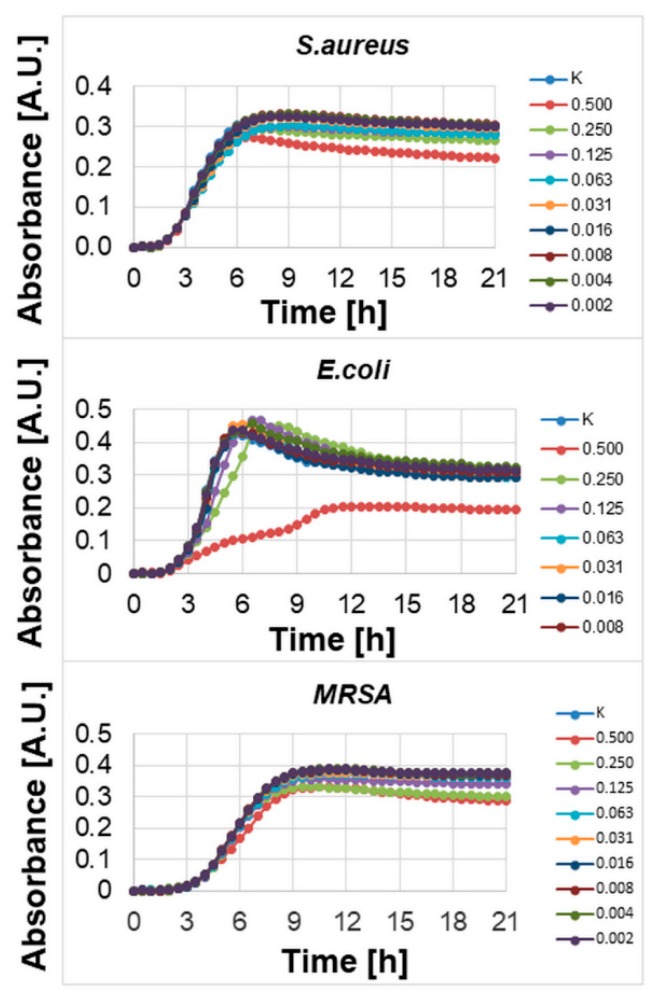
Testing of antimicrobial activity of complex **3** in concentration range 0.002–0.500 mg·mL^−1^ after 21 h of treatment on *S. aureus*, *E. coli,* and *MRSA*.

The growth curves showing on antibacterial activities are depicted in Figure 2.

As can be seen from the curves, there are only low activities on Gram-positive bacteria and the highest concentration used (0.5 mg·mL^−1^) was only able to decrease the growth of *S. aureus*. On the other hand, much higher activity was observed on *E. coli*, where the highest concentration of complex inhibits bacteria growth. Nickel chloride as well as pmdien have no antibacterial activities. When we compare these findings with antibacterial activity of complex **1** [27], it can be concluded that the ligand abb and especially its nickel complex have much better activities against *S. aureus* and comparable antibacterial activity on *E. coli.* One should expect higher antibacterial activity in compounds containing sulfur in the structure, but conformation, i.e., planarity vs. nonplanarity of ligands and possible π–π interactions play a very important role in binding to active sites [33,34]. 

There are various parameters, which could influence the biological properties of a complex. The sphere of complexes **1** and **2** is completed by bisbenzimidazoles. The interaction with DNA is probably based on the intercalation due to the existence of aromatic rings for π–π interaction. There is also a question of orientation of the rings as abb in complex **1** is nearly planar in contrary to the tebb ligand in complex **2**, where coordination is facial-like and there is a longer Ni-S bond distance. Furthermore, ligand abb has one more nitrogen for possible hydrogen bonding with DNA. In contrary, complex **3** has a pmdien ligand coordinated to central atoms. The hydrogens of methyl groups can be used for hydrogen bonding in this case. The helical structure of complex **3**, proved by X-ray study, is predetermined for strong interaction with DNA. The higher antibacterial activity of complex **1** might be also due to the oxidation of the central atom which occurs around −0.2 V; however, it was not observed in the case of complex **3** in the scanned potential window.

### 2.3. Study the Cytotoxic Activity

The cytotoxic activity of complex **3** and ttcNa_3_ were studied on G-361 (human malignant melanoma), HOS (human osteogenic sarcoma), K-562 (human chronic myelogenous leukaemia), and MCF-7 (human breast adenocarcinoma) cancer cell lines. Salt ttcNa_3_ shows no activity against the tested lines. Complex **3** shows cytotoxic activity against all the tested cell lines with the following results: on G-361 the average IC50 value is equal to 31.6 µM, HOS with IC50 = 15.5 µM, K-562 with IC50 = 45.9 µM, and MCF7 with IC50 = 25.1 µM. This is an interesting result, even though, in comparison with IC50 of cisplatin (2.9, 3.0, 4.7, and 10.9 µM) and oxaliplatin (7.1, 6.8, 8.8, and 18.2 µM), complex **3** is less effective, but comparable activity is shown on the MCF7 cell line. It is also of interest that the complex [Cu_3_(pmdien)_3_(μ-ttc)](ClO_4_)_3_ of the same composition as the title complex, except for central atoms, shows no cytotoxic activity [35,36]. The activity of complex **3** can also be explained by its better solubility and finally by its helical structure.

## 3. Materials and Methods

The electrochemical measurements were carried using the electrochemical analyzer 663 VA stand (Metrohm, Switzerland), where a hanging mercury drop (HMDE) was used as the working electrode, a Pt wire served as the counter electrode, and an Ag/AgCl (1 M, KCl) as the reference electrode. The electrochemical measurements were performed in a buffer composed of 1.750 μL H_2_O + 100 μL of the mixture (1 M HCl + 3 M NH_3_). The desired volume of the sample was added to the solution. The CV was applied for the study of the electrochemical behavior of the compounds with parameters as follows: start potential −1.5 V, final potential 0.0 V, scan rate 50 mV·s^−^^1^. Antibacterial properties of complex **3** were studied on *S. aureus* (NCTC 8511), *E. coli* (NCTC 13216), and *MRSA* obtained from the Czech Collection of Microorganisms, Faculty of Science, Masaryk University, Brno, Czech Republic. The antimicrobial effect was determined on the instrument Multiskan EX (Thermo Fisher Scientific, Dreieich, Germany) by absorbance measurement at 600 nm. The cytotoxic activity of complex **3** and ttcNa_3_ was established *in vitro* against four cell lines G-361, HOS, K-562, and MCF-7. The cancer cells were kept in 75 mL tissue culture flasks (TPP) and Dulbecco's modified Eagle's cell culture medium (DMEM) composed of 1 mg·mL^−1^ glucose, 4 mM glutamine, 100 µg·mL^−1^ streptomycin, 10% bovine serum, 100 IU·mL^−1^ penicillin, and 3.7 mg·mL^−1^ Na_2_CO_3_. Approximately 1.25 × 10^−5^ cells·mL^−1^ were poured into 96-well plates (Nunc, 80 µL per well). After incubation (12 h, 37 °C, 5% CO_2_), the tested compounds, in six-fold dilutions, were added (20 µL per well). After incubation of cells (72 h) with the tested compounds, the cells were incubated with calcein AM and the fluorescence of the live cells was measured at 485 nm excitation and 538 nm emission on Fluoroscan Ascent (Labsystems, Vantaa, Finland). IC50 values, the drug concentrations lethal to 50% of the cancer cells, were guessed.

## 4. Conclusions

The redox reaction of the trinuclear nickel complexes was studied. Mainly, the observed redox peaks are attributed to the presence of ttc and nickel(II) ion in the complexes. However, the redox peaks are affected by the spatial structure and composition of the studied complexes. Furthermore, the antibacterial study of the complexes revealed that the presence of an appropriate ligand can induce the antibacterial properties to the trinuclear nickel(II) complex. Interestingly, complex **3** shows a cytotoxicity effect on the tested cell lines, while ttcNa_3_ did not show any cytotoxicity activity. As explained, the type of the central atom as well as the solubility and the helical structure affect the cytotoxic activity of the complex. It must be mentioned that the potential toxicity of ttc containing complexes can be overcome by drug carriers like liposomes [37] or apoferritin [38]. Anyway, further study (in vivo) is necessary to prepare and characterize compounds with potential use in medicinal practice.
